# eLD: entropy-based linkage disequilibrium index between multiallelic sites

**DOI:** 10.1038/s41439-018-0030-x

**Published:** 2018-10-22

**Authors:** Yukinori Okada

**Affiliations:** 1 Department of Statistical Genetics, Osaka University Graduate School of Medicine, Suita, 565-0871 Japan; 2 Laboratory of Statistical Immunology, Immunology Frontier Research Center (WPI-IFReC), Osaka University, Suita, 565-0871 Japan

## Abstract

Quantification of linkage disequilibrium (LD) is a critical step in studies investigating human genome variations. Commonly used LD indices such as *r*^*2*^ handle LD of biallelic variants for two sites. As shown in a previously introduced LD index of *ε*, normalized entropy difference of the haplotype frequency between LD and linkage equilibrium (LE) could be utilized to estimate LD of biallelic variants for multiple sites. Here, we developed eLD (**e**ntropy-based **L**inkage **D**isequilibrium index between multiallelic sites) as publicly available software to calculate *ε* of multiallelic variants for two sites. Application of eLD could dissect complex LD structures among multiple HLA genes (e.g., strong LD among *HLA-DRB1*, *HLA-DQA1*, and *HLA-DQB1* in East Asians). Use of eLD is not restricted to haplotype-based LD; it is also applicable to genotype-based LD. Therefore, eLD enables estimation of *trans*-regional LD of SNP genotypes at two unlinked loci, such as the nonlinear LD between functional missense variants of *ADH1B* (rs1229984 [Arg47His]) and *ALDH2* (rs671 [Glu504Lys]).

Linkage disequilibrium (LD) is defined as the nonrandom distribution of alleles at different loci^1^. Quantitative assessment of LD in a population of interest is an important procedure to conduct fine-mapping of causal variants embedded in the disease risk loci identified by genome-wide association studies (GWAS)^2^. Population-specific features of LD are related to ethnically heterogeneous distributions of single-nucleotide polymorphisms (SNPs)^3^. The most widely used measurements of LD are *r*^*2*^ and *D*′; both values quantify LD between biallelic variants (i.e., SNPs) for two sites, reflecting nonrandom distributions of four haplotypes consisting of pairwise combinations of the alleles. Specifically, *r*^2^ can be interpreted as Pearson’s correlation measurement (*R*^2^) of allele distributions and is known to be proportional to *χ*^2^ values of genotype–phenotype association statistics between two sites^1^. LD values can easily be calculated using publicly available software (e.g., PLINK and vcftools), or using downloaded pre-calculated values from websites (e.g., HaploReg and LocusZoom).

Nothnagel et al.^4^ previously demonstrated that *r*^2^ can also be interpreted as normalized entropy in haplotype frequencies, and introduced a novel LD index named *ε* (see definition in Supplementary Information). *ε* represents the normalized entropy difference of the haplotype frequencies between LD and those expected under the null hypothesis of no LD (i.e., linkage equilibrium [LE]). The value of *ε* ranges between 0 and 1, with larger values indicating stronger LD. Application of *ε* enabled LD quantification of biallelic variants for multiple sites (Fig. 1)^4^, which was effective in selecting tag SNPs free from ambiguous definitions of LD blocks in an unbiased manner^5^.

**Fig. 1:**
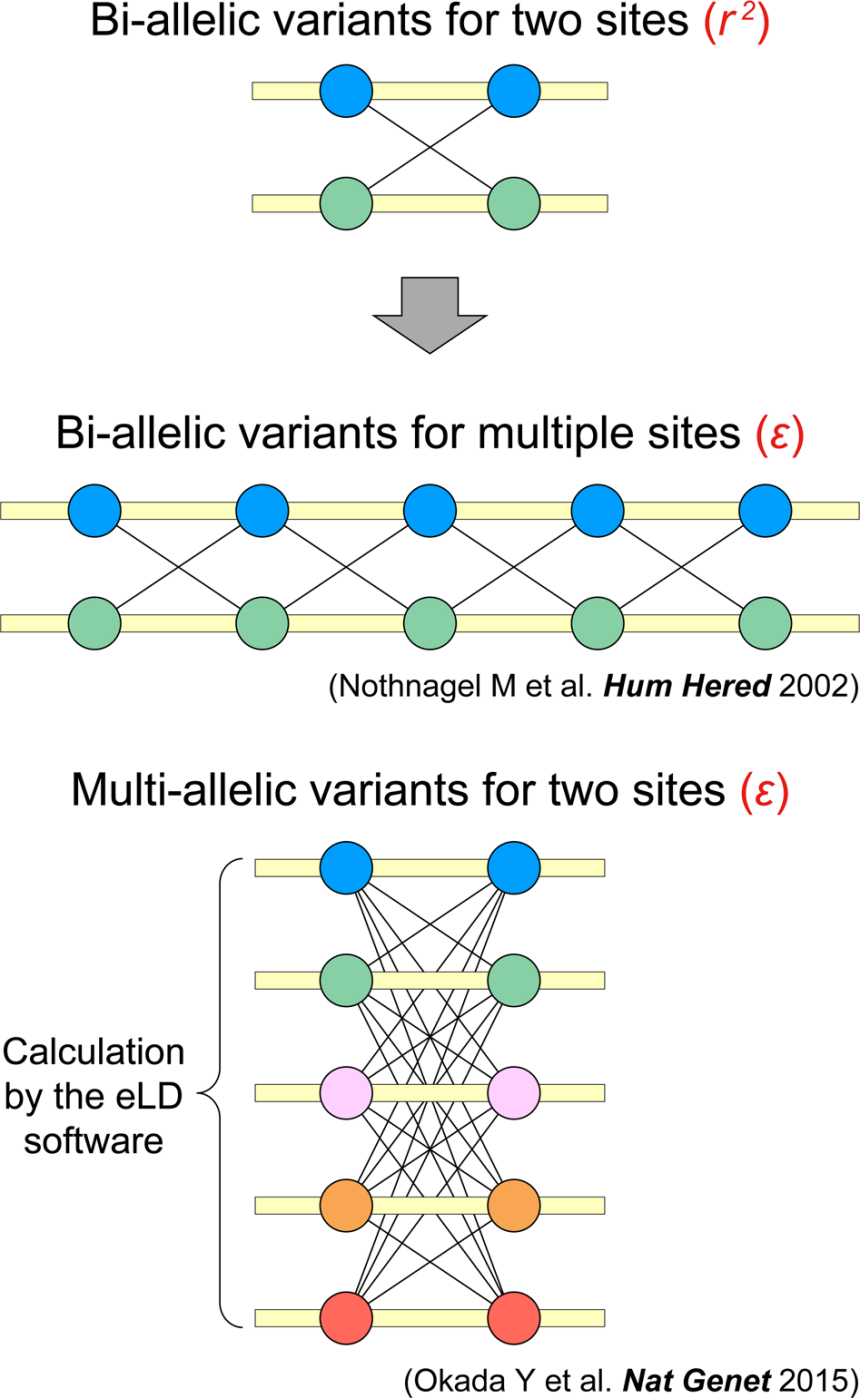
**eLD: entropy-based linkage disequilibrium index between multiallelic sites.** While *r*^*2*^ quantifies LD of biallelic variants for two sites, *ε* quantifies LD of biallelic variants for multiple sites^4^ or multiallelic variants for two sites^6^. We developed eLD (**e**ntropy-based **L**inkage **D**isequilibrium index between multiallelic sites) as publicly available software to calculate *ε* of multiallelic variants for two sites (see the software URL)

We have recently extended *ε* to further quantify LD of multiallelic variants for two sites as described elsewhere (Fig. 1)^6^. Here, we developed eLD (**e**ntropy-based **L**inkage **D**isequilibrium index between multiallelic sites) as publicly available software to calculate the *ε* of multiallelic variants for two sites (see the software URL). Various multiallelic variants exist with important clinical impacts in terms of genotype–phenotype associations. Of these, polymorphisms of human leukocyte antigen (HLA) genes in the major histocompatibility (MHC) locus have a wide spectrum of risk for a variety of human diseases. While elucidation of the complex LD structure of HLA genes has been challenging, application of *ε* clearly identified hidden LD relationships among the HLA genes^6^. For example, we observed relatively strong LD between *HLA-C* and *HLA-B*, among *HLA-DRB1*, *HLA-DQA1*, and *HLA-DQB1*, and between *HLA-DPA1* and *HLA-DPB1* (*ε* *>* 0.15; calculated using 4-digit classical alleles of a subset of the East Asian subjects [*n* = 300] enrolled in the original studies^6,7^; Fig. 2). Since estimation of the haplotype frequency could be biased when its distribution is sparse, an option to combine the alleles with frequencies lower than the defined threshold (0.05 in default settings) into a single dummy allele is implemented in eLD.

**Fig. 2:**
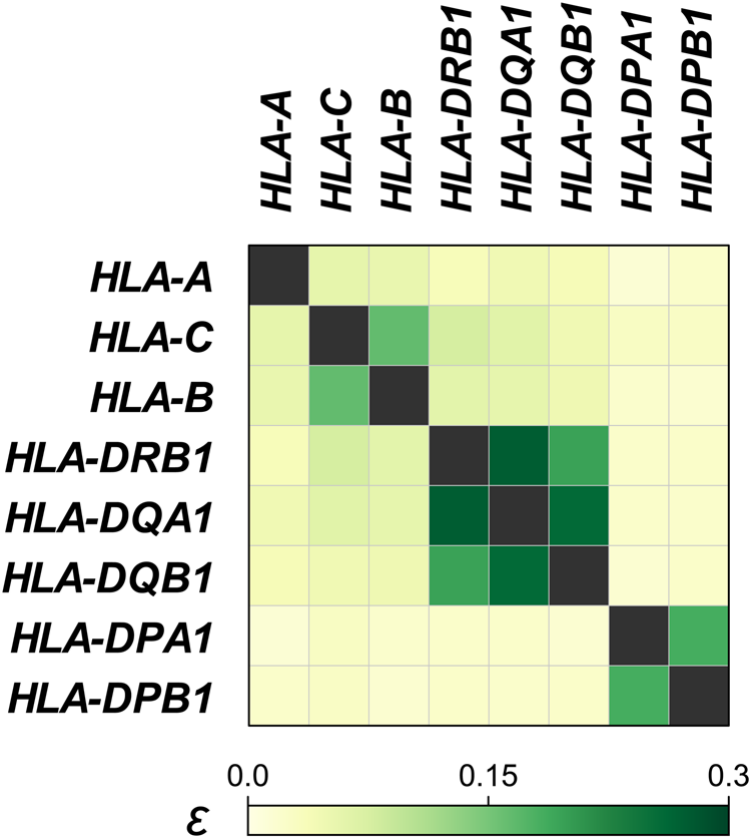
**Pairwise quantification of LD among the classical HLA gene variants.** Pairwise LD index of *ε* among the 4-digit alleles of the classical HLA genes were evaluated using eLD. Phased HLA alleles were obtained from a subset of the East Asian subjects (*n* = 300) of the original studies^6, 7^. Strong LD between *HLA-C* and *HLA-B*, among *HLA-DRB1*, *HLA-DQA1*, and *HLA-DQB1*, and between *HLA-DPA1* and *HLA-DPB1* was observed (*ε* > 0.15)

One of the novel features of eLD is to empirically estimate a value of *ε* in a null hypothesis of LE (= *ε*__NULL_). Additionally, it also calculates the *ε* actually observed in a given data set ( = *ε*__Observed_). eLD calculates *ε*__NULL_ based on a permutation approach. By randomly shuffling connections of the alleles between the two sites, *ε*__NULL_ is estimated as the mean value of *ε* obtained in each iteration step (×1000 iterations in default settings). Since the baseline value of *ε*__NULL_ depends on the number of alleles in each site, calculation of *ε*__NULL_ as well as *ε*__Observed_ would help to evaluate the relative strength of LD relationships at the observed sites.

Another feature of the software is that application of eLD is not restricted to haplotype-based LD; it is also applicable to genotype-based LD. Using eLD, one can estimate LD between loci where phasing of the haplotypes is theoretically difficult. As an illustrative example, we estimated *trans*-regional LD in two unlinked loci: *ADH1B* at 4q23 and *ALDH2* at 12q24. *ADH1B* and *ALDH2* harbor well known functional missense variants at rs1229984 (Arg47His) and rs671 (Glu504Lys), respectively. Both of these SNPs have pleiotropic effects on a number of human complex traits, including dietary habits. Studies investigating natural selection pressure identified strong significant positive selection on these missense variants in Japanese or other East Asian populations, which was closely linked to geographical heterogeneity in allele frequency spectra of these SNPs even within a single population^8^. Here, using eLD, we calculated *ε* to estimate *trans*-regional LD between rs1229984 and rs671 (Fig. 3). We obtained genotypes for these SNPs from East Asian subjects within the 1000 Genomes Projects (*n* = 504, phase 3 version 5), and found a high *ε*__Observed_ value (=0.0053) when compared to *ε*__NULL_ (= 0.0024). As expected from natural selection pressure on these variants^8^, rs1229984AA-rs671AA genotypes and rs1229984GG-rs671GG genotypes had increased frequencies compared to those variants in LE (≥1.21-fold), while rs1229984GG-rs671GA genotypes had decreased frequencies (0.58-fold) compared to those variants in LE. While Pearson’s correlation between genotypes can also evaluate *trans*-regional LD, nonlinear relationships of genotypes (such as the reduced frequency of rs1229984GG-rs671GA) would not have been reflected with this measurement.

**Fig. 3:**
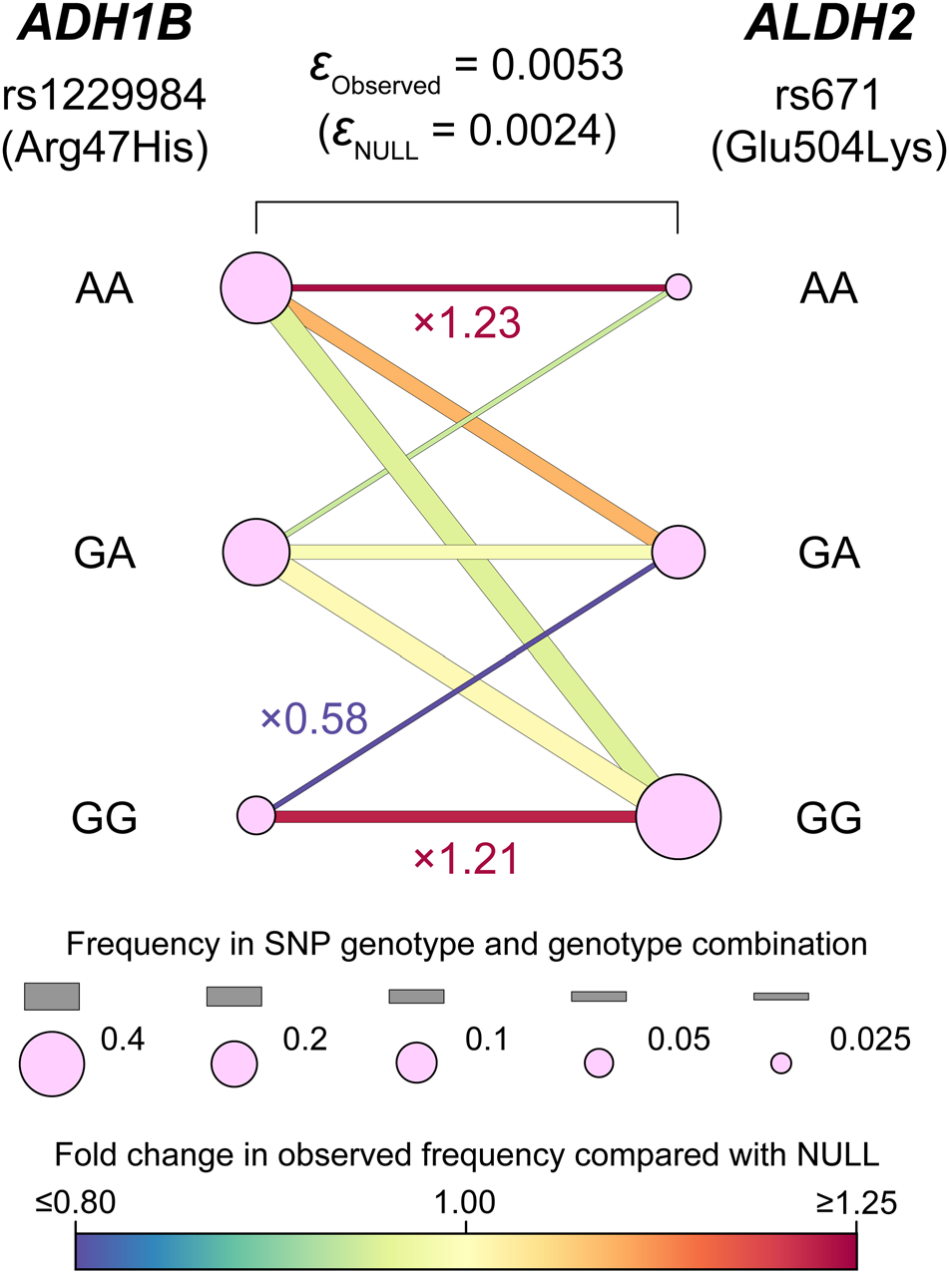
***Trans*-regional LD between functional missense variants of** ***ADH1B*** **and** ***ALDH2***. eLD can quantify genotype-based LD as well as haplotype-based LD and thus can estimate *trans*-regional LD between two unlinked loci without haplotype phasing. LD between genotypes of the two functional missense variants of *ADH1B* (rs1229984 [Arg47His]) and *ALDH2* (rs671 [Glu504Lys]) was assessed by using eLD. Observed *ε* (= *ε*__Observed_) and *ε* expected under the null hypothesis of LE (= *ε*__NULL_) are indicated. Frequencies of the genotypes and genotype combinations are visualized as in the legend. We observed nonrandom and nonlinear distribution of the genotypes between the variants (i.e., reduced frequency in rs1229984GG-rs671GA)

In summary, we developed software, which we named eLD, that quantifies the entropy-based LD index of *ε* in multiallelic variants for two sites, such as LD between highly polymorphic HLA genes. eLD also enables estimation of *trans*-regional LD of SNP genotypes, such as functional variants of *ADH1B* and *ALDH2*. We note that normalized entropy has increased the potential to dissect complex dependencies among human genome variations (e.g., Y-chromosomal short tandem repeat [STR] marker selection^9^), and development of additional methodology should be warranted.

## Software availability

eLD is freely available at http://www.sg.med.osaka-u.ac.jp/tools.html with example data sets.

## Electronic supplementary material

Supplementary Information
