# Supplementary material for: eLD: entropy-based linkage disequilibrium index between multiallelic sites
Source: Hum Genome Var. 2018 Oct 22;5:29. doi: 10.1038/s41439-018-0030-x (PMC6197273; doi:10.1038/s41439-018-0030-x)
Supplement: Supplementary file 1 — Supplementary Information [file 41439_2018_30_MOESM1_ESM.docx]

**Supplementary Information**

**eLD: entropy-based Linkage Disequilibrium index between multi-allelic sites**

Correspondence to Yukinori Okada (yokada@sg.med.osaka-u.ac.jp)

**Definition of *ε***

*ε* represents normalized entropy difference of the haplotype frequencies between LD (linkage disequilibrium) and those expected under null hypothesis of no LD (i.e., linkage equilibrium [LE]).

Let *p*_i_ be an observed frequency of the *i*-th haplotype which consists as a combination of each allele of each site of interests. Let *q*_i_ be an expected frequency of the *i*-th haplotype in LE, which can be estimated as a product of observed frequencies of the alleles harbored in the haplotype. Entropy of the haplotype frequencies in LD (= *S*_LD_) and LE (= *S*_LE_) are represented as

$$S_{\text{LD}}=-\sum_{i} p_{i}\log(p_{i})$$

and

$S_{\text{LE}}=-\sum_{i} q_{i}\log(q_{i})$,

respectively.

Then, *ε* is defined as

$\varepsilon=\frac{\Delta S}{S_{LE}}=\frac{S_{LE}-S_{LD}}{S_{LE}}=1-\frac{S_{LD}}{S_{LE}}$.

Detailed definition and theoretical backgrounds of *ε* are described elsewhere^1,2^.

**References**

1. Nothnagel, M. et al. Entropy as a measure for linkage disequilibrium over multilocus haplotype blocks. *Hum. Hered.* **54**, 186-198 (2005).

2. Nothnagel, M. & Rohde, K. The effect of single-nucleotide polymorphism marker selection on patterns of haplotype blocks and haplotype frequency estimates. *Am. J. Hum. Genet.* **77**, 988-998 (2005).
